# Does muscle fatigue change motor synergies at different levels of neuromotor control?

**DOI:** 10.3389/fnhum.2024.1519462

**Published:** 2025-01-07

**Authors:** Michał Pawłowski, Mariusz P. Furmanek, Grzegorz Juras

**Affiliations:** ^1^Institute of Sport Sciences, Department of Human Motor Behavior, Academy of Physical Education, Katowice, Poland; ^2^Department of Physical Therapy, University of Rhode Island, Kingston, RI, United States

**Keywords:** fatigability, hierarchy control, EMG, force, upper limb coordination

## Abstract

We investigated the effects of static and dynamic fatigue on motor synergies, focusing on their hierarchical control. Specifically, we examined whether changes in fatigue influence the central nervous system’s ability to preserve movement stability. In addition to exploring the direct impact of fatigue on motor synergies, we also analyzed its effects at two distinct levels of hierarchical control, aiming to elucidate the mechanisms by which fatigue alters motor coordination and stability. Thirteen healthy, young and right-handed male participants took part in the study. Participants performed a bilateral accurate force production task under static and dynamic fatigue conditions at 30% of maximal voluntary contraction level with elbow flexors. Muscle activity level were collected from five muscles of each limb: biceps brachii, brachialis, brachioradialis, flexor carpi radialis, and flexor carpi ulnaris. The results revealed distinct effects of fatigue on isometric force production in the elbow joint tasks. On the higher level of hierarchy control of synergies, there were non-significant effects of different types of fatigue on movement performance, however, on the lower level we observed a strong effect of fatigue on forming motor synergies. There was no significant difference between the type of applied fatigue protocol on force and muscle activity data, nevertheless, the contribution of involved muscles to the task has changed. Our findings indicate that the central nervous system employs specific strategies to counteract fatigue and preserve movement stability during performance. However, the precise mechanisms by which variability at lower levels of hierarchical control influence higher levels remain unclear, highlighting a critical gap in our understanding of motor coordination under fatigue. Future studies should explore how these interactions across hierarchical levels contribute to movement stability under different fatigue conditions.

## 1 Introduction

The primary goal and function of the central nervous system (CNS) is to perform coordinated and effective movements (motor tasks) that are adequate for the current conditions. In addition to aging and motor learning processes, the most common changes in human motor behavior are caused by fatigue. Despite the effects of fatigue, the CNS has to ensure a stable and safe movement performance, not only for routine daily activities but also during professional duties and other motor behaviors. The effects of fatigue on athletic training (strength and endurance) are relatively well known (Behm et al., 2021; Vieira et al., 2022; Alix-Fages et al., 2023); however, explicit conclusions regarding its impact on movement control remain elusive. Despite numerous studies, questions remain unanswered regarding how a football (or basketball) player reacts during extra time or at the end of an intense match, and how the precision of a player’s movement or ball control changes in these situations. How is it possible that after prolonged movement performance, players are still able to throw a ball successfully to the basket? What enables CNS for effective movement execution despite of existing fatigue?

Numerous studies show the remarkable capacity of the CNS to counteract the impact of fatigue on movement performance (Jones and Hanson, 1971; Bonnard et al., 1994; Madeleine et al., 2002). Despite relatively small differences in the final effect of the goal-directed movement (e.g., ball catching or throwing to the target), the CNS seems to use variability among the involved structures of the neuromusculoskeletal system to ensure effective performance, especially for multi-joint actions. This phenomenon was first observed by Bernstein (1967) and formulated as the classical problem of motor redundancy. Contemporary solutions to Bernstein’s problem (Latash et al., 2007; Latash and Zatsiorsky, 2016; Latash, 2021) place motor synergies as a promising neurophysiological mechanism to solve redundant features in movement control. Synergies (defined in at least three different variations in the motor control area) allow CNS to stabilize the specific variables responsible for a given motor task execution [e.g., the specific organization of muscle activities stabilizes the trajectory of the upper limb during a throwing movement (Hasanbarani and Latash, 2020)]. Synergies are the coordinated effort of the elements of the musculoskeletal system responsible for movement execution (Latash and Zatsiorsky, 2016). In this approach, the synergistic contractions of muscles produce body movements that simultaneously stabilize the generated forces to execute the specific motor task. The introduced notion of motor synergies assumes the specific hierarchical organization of their control with at least two levels (Latash, 2012). The upper levels of the control system provide the essential requirements for the movement performance (higher level of the hierarchy) in relation to crucial lower levels (such as motor units), which are responsible for movement production features (lower level of the hierarchy) (Latash et al., 2008; Latash, 2021; Latash et al., 2023). The above mentioned stabilization process is compromised by various disturbing stimuli with fatigue emerging as the most prevalent factor significantly impacting movement control (Forestier and Nougier, 1998; Jaric et al., 1999). Due to this complexity, there is no single unanimously accepted definition of fatigue (Gandevia, 2001; Hunter et al., 2004; Behrens et al., 2023). For the purposes of this study, we define fatigue as a decrease in the ability of the neuromuscular system to generate maximum muscle force or power (Bigland-Ritchie et al., 1986; Enoka and Stuart, 1992; Enoka et al., 2011). To assess the effects of fatigue, the concept of fatigability was introduced, which permits an objective analysis of the decrease in generated forces over a given time (Kluger et al., 2013; Hunter, 2018; Paris et al., 2022). Fatigability is defined as a decrease in the ability of the CNS to generate forces or to reach the accepted threshold of failure during the movement performance with submaximal efforts. Nonetheless, the whole phenomenon is strongly task-specific and does not imply a single major mechanism underlying its effects (Enoka and Duchateau, 2008, 2016; Hunter, 2009).

Our study, investigated three main questions: First, does successful motor task performance persist despite fatigue? Previous studies have reported execution of successful movements after/during fatigue occurs without changes to movement kinematics (Côté et al., 2002; Huffenus et al., 2006; Cowley and Gates, 2017). These studies suggest that CNS has a way to override all negative effects of fatigue. In contrast to the above studies, our study tests this prediction in an isometric force production task. We hypothesize that despite fatigue, participants will perform the given motor task successfully. To address this hypothesis, we quantified the force magnitude before and after applying the fatigue protocol. Furthermore, is there an association between different levels of hierarchical control of motor synergies after fatigue? If CNS performs movement successfully on a higher level of control after fatigue, what will happen on the lower level? Earlier studies in the literature (Gorniak et al., 2007a, 2007b; Singh et al., 2012) suggested difficulties in effective movement control simultaneously on many levels of the applied hierarchical scheme of control. Previous studies have shown lack of changes in the upper (performance) level of control, in contrast to the lower level (Lucidi and Lehman, 1992; Turpin et al., 2011; Ortega-Auriol et al., 2018; Hajiloo et al., 2020). Furthermore, after executing the fatigue protocols, a significant decrease in particular joint motion was observed and this drop was associated with the increase of variability of motion in other joints (Côté et al., 2002; Gates and Dingwell, 2008). Therefore, we hypothesize that different types of fatigue will affect the hierarchical organization of movement control. We predict that fatigue will have significant changes on the lower level of the hierarchy which concerns muscle activity during applied force production tasks. Which in turn, will lead to the formation of distinct motor synergies at this level (Hypothesis #1).

Second, whether the type of muscle fatigue (static vs. dynamic) will affect the force production task? Despite previous studies with various levels of analysis (Al-Mulla et al., 2011; Krüger et al., 2018; Paris et al., 2022), changes in movement performance after different types of fatigue are still not fully understood. Little is known about the consequences of fatigue as a result of different types of muscle contraction. Usually, the effects of fatigue protocols performed in static or dynamic conditions are studied separately. Hence, there is a relatively small number of studies where this paradigm has been investigated within the same group of participants. While some investigators emphasize the importance of the type of muscle contraction for effective movement control (Nussbaum, 2001; Lanza et al., 2004), others suggest contradictory conclusions (Christensen et al., 1995; Sogaard, 1995; Arias et al., 2015). Thus, the results from these studies are inconsistent. We hypothesize that the type of fatigue will affect motor task performance in the force production task (Hypothesis #2).

Furthermore, we hypothesize that the fatigue will enable the CNS to the recruitment a different set of muscles to achieve successful task performance. We investigated the possibility of the involvement of additional degrees of freedom by CNS in force production tasks. Closer examination of previous results (Pawłowski et al., 2021) suggests, that CNS will use the wrist flexors, which were supposed to be inactive (per instruction) in such motor tasks in healthy populations, during the force production task with the elbow joint. Perhaps, CNS will adapt by incorporating alternative muscle groups (not only limited to the elbow flexors but also including the wrist flexors), allowing for effective completion of the motor task (Hypothesis #3).

## 2 Methods

### 2.1 Participants

Thirteen male participants took part in this study (age 19.7 ± 1.49 years old, body height 180.5 ± 6.85 cm and body mass 80.2 ± 10.75 kg). All participants were right-handed, according to the results of the Edinburgh Handedness Inventory survey (Oldfield, 1971). All participants were healthy and reported no known neurological disorders or upper extremity injuries. All participants signed consent forms approved by the Institutional Review Board (7/2013). All applied procedures were conducted with agreement to the Declaration of Helsinki (World Medical Association, 2013).

### 2.2 Apparatus

**Figure 1:**
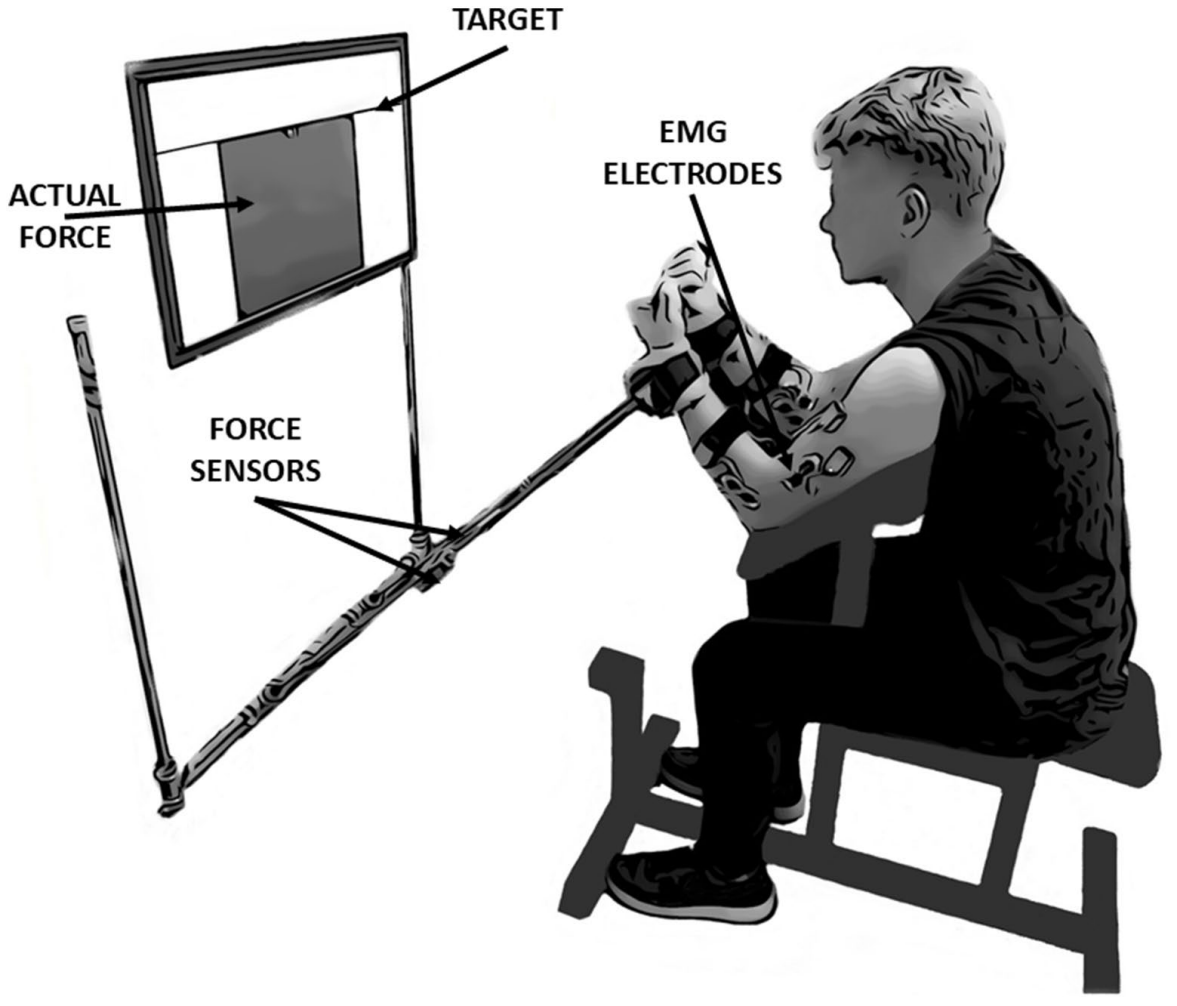
The experimental setup and initial position of the participant. The study was based on performing simultaneous isometric flexion movement in both elbow joints. During the measurements, participants had continuous access to feedback displayed on the screen in front of their eyes. In this study, the kinetic and electromyographic variables were analyzed: force data derived from force sensors located in the middle of the arms of the presented measurement system, whereas EMG data derived from wireless sensors located on five muscles of each upper limb: Biceps brachii, Brachialis, Brachioradialis, Flexor Carpi Radialis and Flexor Carpi Ulnaris.

In the present study, kinetic (custom dynamometer) and electromyographic (EMG) (DTS Noraxon, United States) data were collected. Self-adhesive hydrogel electrodes (Kendall, 30 mm × 24 mm) were used to measure the muscle activity level of five muscle bellies simultaneously on a dominant and non-dominant upper extremity: the biceps brachii (BB), brachialis (BR), brachioradialis (BRD), flexor carpi radialis (FCR) and flexor carpi ulnaris (FCU). Electrodes were placed according to the SENIAM recommendations (Hermens et al., 2000) and anatomical EMG guidelines (Perotto, 2005). The custom dynamometer consists of two one-axis force sensors (model 060-P665-01, Honeywell, United States), placed in the middle of two steel arms of the experimental setup. Each of the force sensors measured forces generated by upper limbs separately. A 32-inch feedback screen (model UE32M5622, Samsung) was located on the wall of the laboratory 0.6 m from the subject’s head at eye level (Figure 1). The resolution of the screen was set to 1,920 × 1,080 pixels. Kinetic and EMG data were synchronized using Noraxon software (MyoResearch, ver. 1.08.17). Both signals were sampled at 1500 Hz.

### 2.3 Experimental procedure

**Figure 2:**
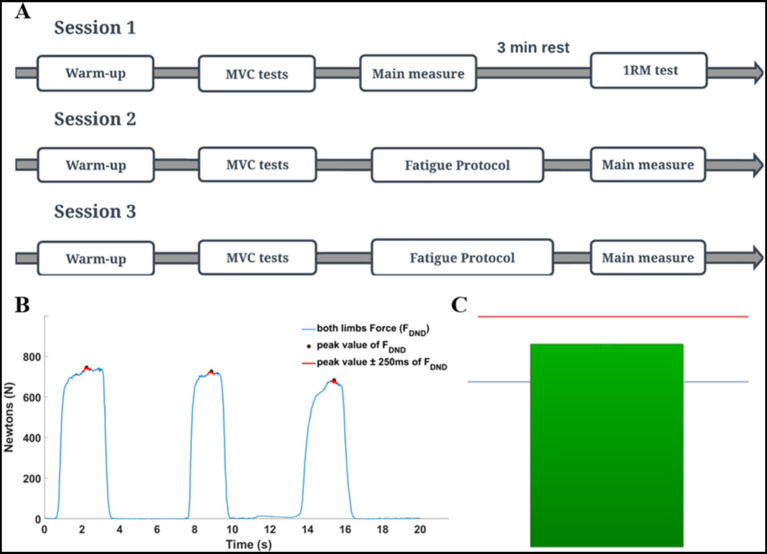
The details of the experimental procedure. **(A)** General scheme of all sessions of the study. **(B)** Maximal voluntary contraction (MVC) was calculated based on a global mean value of three consecutive repetitions. Hence, for each repetition, the mean value of force was extracted from the ±250 ms time interval window in relation to the peak value. MVC test of elbow flexors was always performed in an isometric way with using the dynamometer. **(C)** Schematic of the feedback given to participants during the fatigue protocol (red line indicates the participant target in the fatiguing static task (85% of MVC); blue line indicates the threshold (signal) for the experimenter (70% of MVC) to the end of the fatiguing protocol). When participants could not maintain force to the red line, they were strongly encouraged by the experimenter to do so. If they were not able to maintain force level for 5 consecutive sec. The fatigue trial was over. The next trial was started after 5 s. The protocol was terminated when if participants could not produce force higher than the blue line in the subsequent trial.

All data recordings consisted of three consecutive laboratory sessions (1-baseline, 2-3-fatigue conditions) with 48-h rest intervals between sessions. Each session lasted approximately 60 min. Conditions with fatigue were randomized among the participants (Figure 2A). All tasks in the present study were performed by using the Scott bench. During the measurements, participants were seated on a bench and placed their forearms into the handles of the custom dynamometer. The handles were placed below the wrist joints. The distance between the bench and the feedback screen was adjusted to achieve a 90-degree angle between dynamometer arms and participants’ forearms as well as in elbow joints. Chest and arms were lying on the bench support, as per Evans (2007). This position permitted the execution of isometric force production tasks in flexion movement at the elbow joints simultaneously (Figure 1).

Before data collection, participants performed a warm-up and maximal voluntary contraction test (MVC). Elbow flexion one maximal repetition testing (1RM) was performed by using a barbell curl exercise at the end of the first session. An applied warm-up protocol was created based on Willardson and Burkett (2005) and Pereira et al. (2013) with modifications. Depending on the applied fatigue protocol, two kinds of warm-ups were used: in a static condition, the warm-up was performed with an isometric force production task by using the dynamometer, then in a dynamic condition with the barbell curl exercise on Scott’s bench. During each session, the MVC test was performed separately for elbow flexors and wrist flexor muscles. The MVC test was based on three maximal voluntary contractions of the flexor muscles in 3-s time intervals (Figure 2B). After the MVC test, participants performed the given fatigue protocol and immediately after, the force production task was performed.

### 2.4 Fatigue protocols

The static fatigue protocol consisted of producing and maintaining the 85% MVC value in the isometric force production task in the elbow flexors for as long as possible. During the measurements, real-time feedback on the actual force (sum of the forces produced by dominant and non-dominant limbs) was displayed on the screen in front of the participant. The participant’s task target (85% MVC) was represented as a red line on the screen (Figure 2C). When the generated force decreased below the target, the experimenter asked the participant to reach the target again. If the participant was not able to reach the target (after additional cueing) within time of 5 s, the one repetition of the fatigue task was terminated. The next repetition of the fatigue task began 5 s after the prior trial. The static protocol of fatigue was over when a participant, in the one of the subsequent repetition of the fatiguing task, was unable to produce force at the level of 70% of the MVC test {blue line on the screen [participants were unaware of the meaning of this marker (Figure 2C)]}.

The dynamic fatigue protocol involved performing exercise based on a barbell curl exercise on Scott’s bench. Participants performed the elbow flexions at the level of 85% of 1RM synchronized to a metronome (2 s for the eccentric and 2 s for the concentric phase of the elbows flexion). A repetition of the dynamic fatigue task was finished when the participant was not able to perform the flexions in the set time interval (also with additional cueing from experimenter). The dynamic protocol of fatigue was terminated when a participant in the one of the subsequent repetition of the fatiguing task was not able to execute five proper (instructed) elbow flexions. The both fatigue protocols were implemented by Maluf and Enoka (2005) with modifications.

### 2.5 Outcome measures and data processing

**Figure 3:**
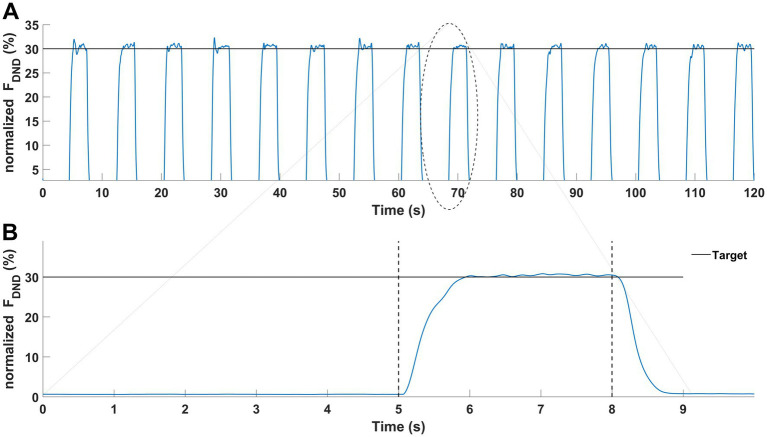
Graphical representation of the force production task. Results of a representative participant. **(A)** Force production task during one condition (15 trials). **(B)** Force production task in a single trial was performed with two phases: no force (first 5-s) and force phase (last 3-s).

Immediately, after finishing the fatigue protocols, the force production task was performed. Participants executed 15 consecutive trials (Pawłowski et al., 2021) of force production in elbow joints at the level of 30% of the MVC test. Each trial consisted of two phases lasting 8 s in total (Figure 3A). Participants generated the force only during the second phase of each trial. The representative measurement of one participant is presented in Figure 3B. All signals were processed offline using Matlab software (ver. 2024A, MathWorks, United States). Force data was low-pass filtered at 10 Hz and EMG data was filtered at 20–360 Hz with a 4th-order digital Butterworth filter. EMG data were detrended, rectified and a moving 100-ms window RMS algorithm was applied. Then both signals were normalized to the results of the MVC tests. Next, the force and EMG data cycles were extracted (phase 2 of the main task) and then the middle 1-s time interval of each cycle was chosen for further analysis. Finally, force and EMG data of extracted one-second time intervals were averaged. The selected variables in the statistical analyses included: mean values of the sum of generating forces by both upper limbs (*F*_DND_), the force generated by the dominant (*F*_D_) and non-dominant upper limb (*F*_ND_), the difference of force between upper limbs (*F*_DIFF_), the committed force error in relation to the target (*F*_ERR_) and the mean values of the normalized EMG data from all 10 chosen muscles.

### 2.6 Statistical analysis

All results are reported as mean and standard deviation. First, to test Hypotheses #1 and #2, we tested the effects of fatigue of static and dynamic type on force magnitude as well as individual muscle activity level of elbow flexors (in dominant and non-dominant upper extremities) with one-way 1 × 3 rmANOVA (repeated measurements, one factor: fatigue type: baseline, static, dynamic). To explore Hypothesis #3, we analyzed the level of muscle activity of elbow flexors, separately for each upper extremity. For this purpose we used separate 1×3 ANOVAs (three muscles: BB, BR and BRD) to determine the differences between analyzed muscles for all fatigue conditions (baseline, static, and dynamic). Hypothesis #4 was tested using similar 1 × 5 ANOVAs analyses on EMG data with additional data from wrist flexors. The basic assumptions of these analyses were checked using the Shapiro-Wilks (normal distribution of data) and Levene’s test (homogeneity of variance). Significant differences between specific measurements were examined with Bonferroni *post hoc* testing. All statistical analyses were performed using Statistica 13 (StatSoft, United States) software at the applied alpha level of 0.05. All effect sizes were reported as partial eta-squared (*η*_p_^2^).

## 3 Results

### 3.1 Effects of fatigue on force and EMG activity

**Figure 4:**
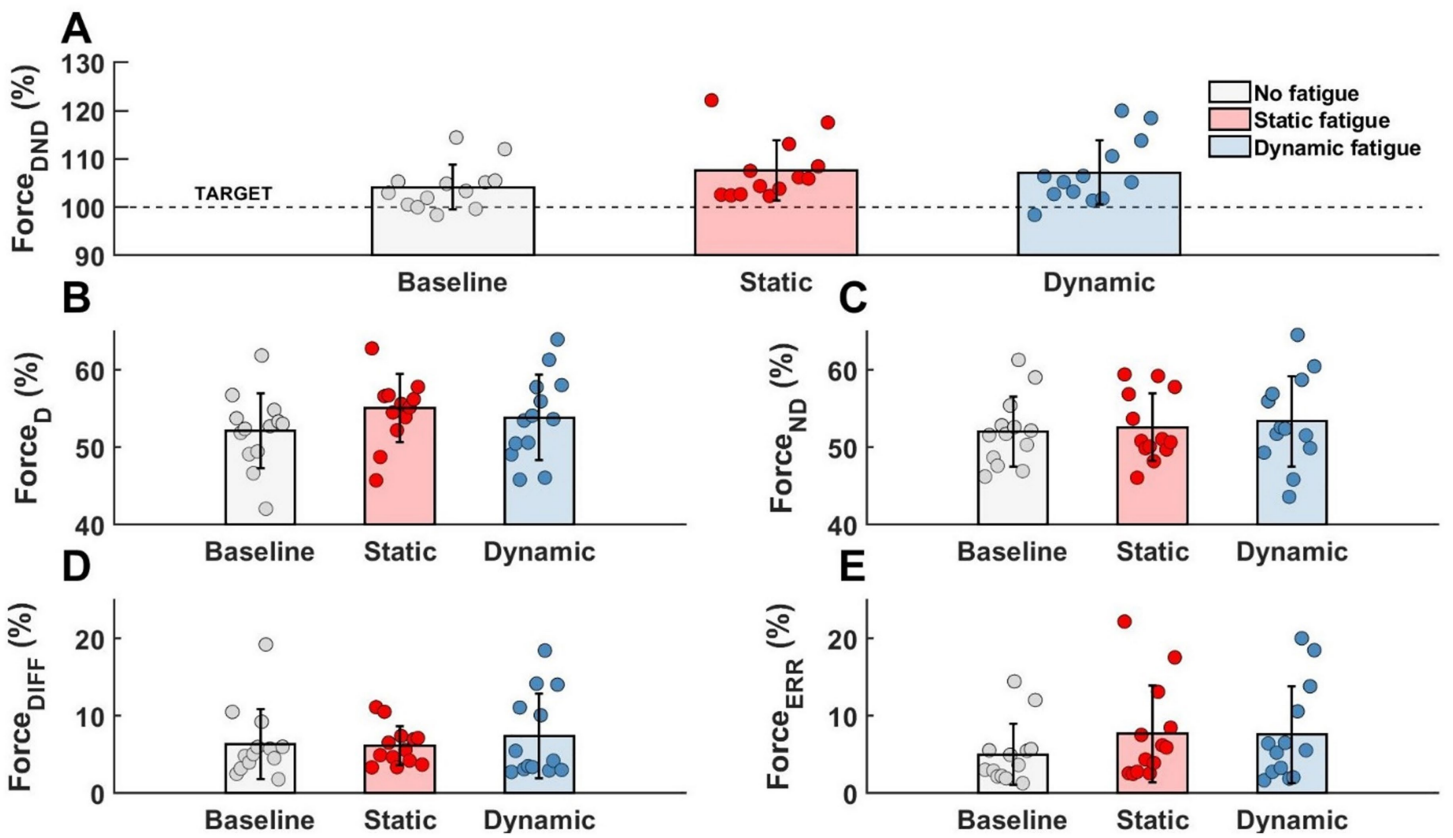
Lack of the significant effects of different types of fatigue on force level analysis. **(A)** Forces generated by both limbs, **(B)** force generated by dominant limb, **(C)** force generated by non-dominant limb, **(D)** difference between limbs, **(E)** participants error.

Regardless of the specific fatigue protocol, we reported an increase in force level in *F*_DND_, *F*_D_ and *F*_ND_. Furthermore, the contribution of the force between limbs (*F*_DIFF_) was changed and participants were less accurate during the force production task (*F*_ERR_). However, any above mentioned changes were not statistically significant (Figure 4).

**Figure 5:**
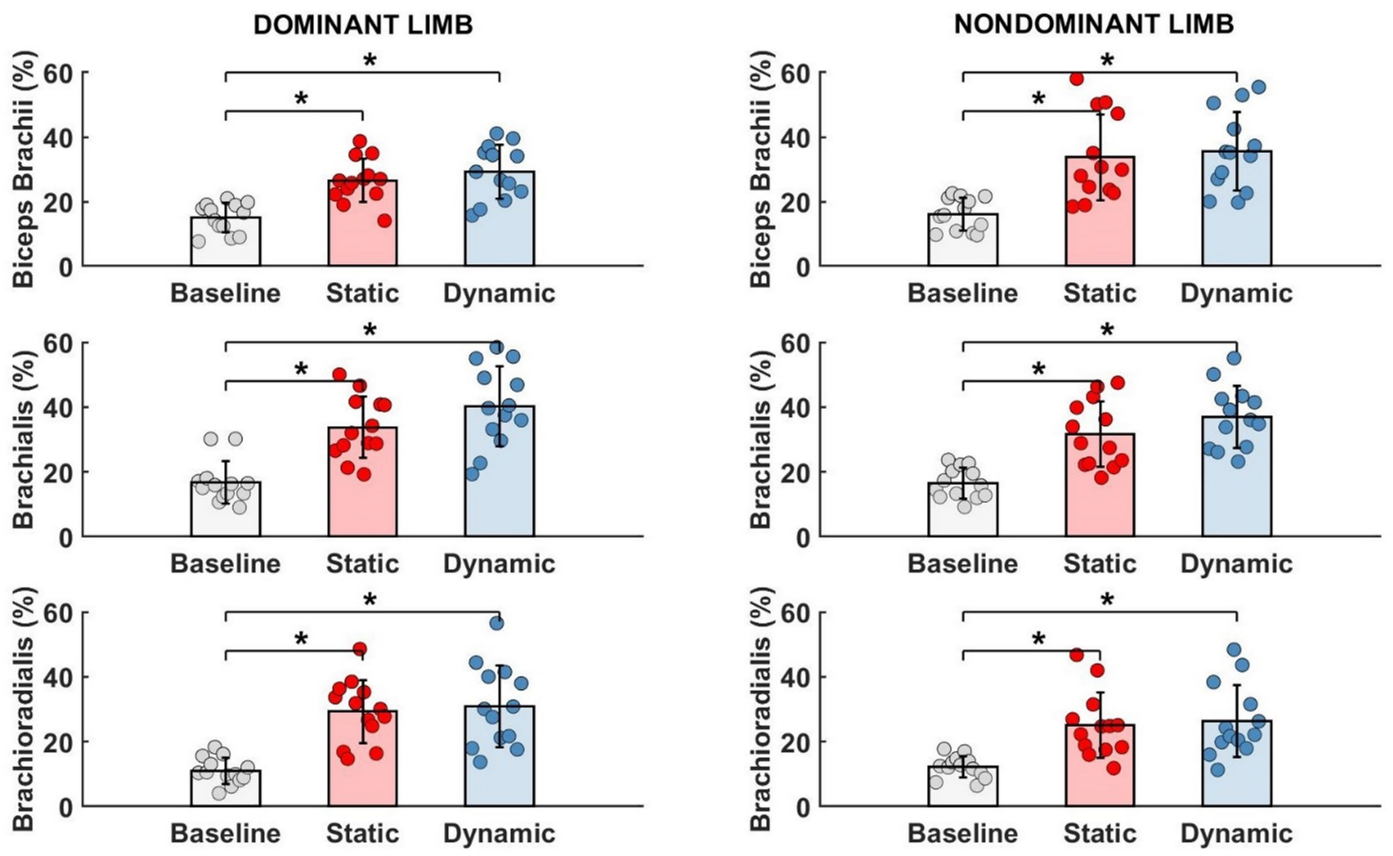
Significant impact of different types of fatigue on elbow flexors EMG activity. * - *p*<0.05.

As shown in Table 1, the muscle activity significantly increased in all elbow flexors for the dominant as well as in the non-dominant limb. Regardless of the type of fatigue protocol used, the activity of all analyzed muscles increased by more than 100%. Fatigue in dynamic conditions seems to be more challenging for the participants due to achieving higher values of the EMG signal. However, this tendency and difference between both fatigue conditions were not statistically significant (Figure 5).

**Table 1:** Main effects of rmANOVA analysis of fatigue effects on muscle activation space.

	Dominant Limb			Non-dominant Limb		
Muscle	Main effect of fatigue	Impact of static fatigue	Impact of dynamic fatigue	Main effect of fatigue	Impact of static fatigue	Impact of dynamic fatigue
BB	*F*_(2,24)_ = 18.508 *p* < 0.001 *η*_p_^2^ = 0.607	*p* < 0.001	*p* < 0.001	*F*_(2,24)_ = 22.208 *p* < 0.001 *η*_p_^2^ = 0.649	*p* < 0.001	*p* < 0.001
BR	*F*_(2,24)_ = 22.352 *p* < 0.001 *η*_p_^2^ = 0.651	*p* < 0.001	*p* < 0.001	*F*_(2,24)_ = 32.876 *p* < 0.001 *η*_p_^2^ = 0.736	*p* < 0.001	*p* < 0.001
BRD	*F*_(2,24)_ = 28.991 *p* < 0.001 *η*_p_^2^ = 0.707	*p* < 0.001	*p* < 0.001	*F*_(2,24)_ = 12.389 *p* < 0.001 *η*_p_^2^ = 0.508	*p* = 0.001	*p* < 0.001

*ηp2, effect size explained with partial eta-square; BB, biceps brachii; BR, brachialis; BRD, brachioradialis.*

### 3.2 Effects of fatigue on motor synergies

**Figure 6:**
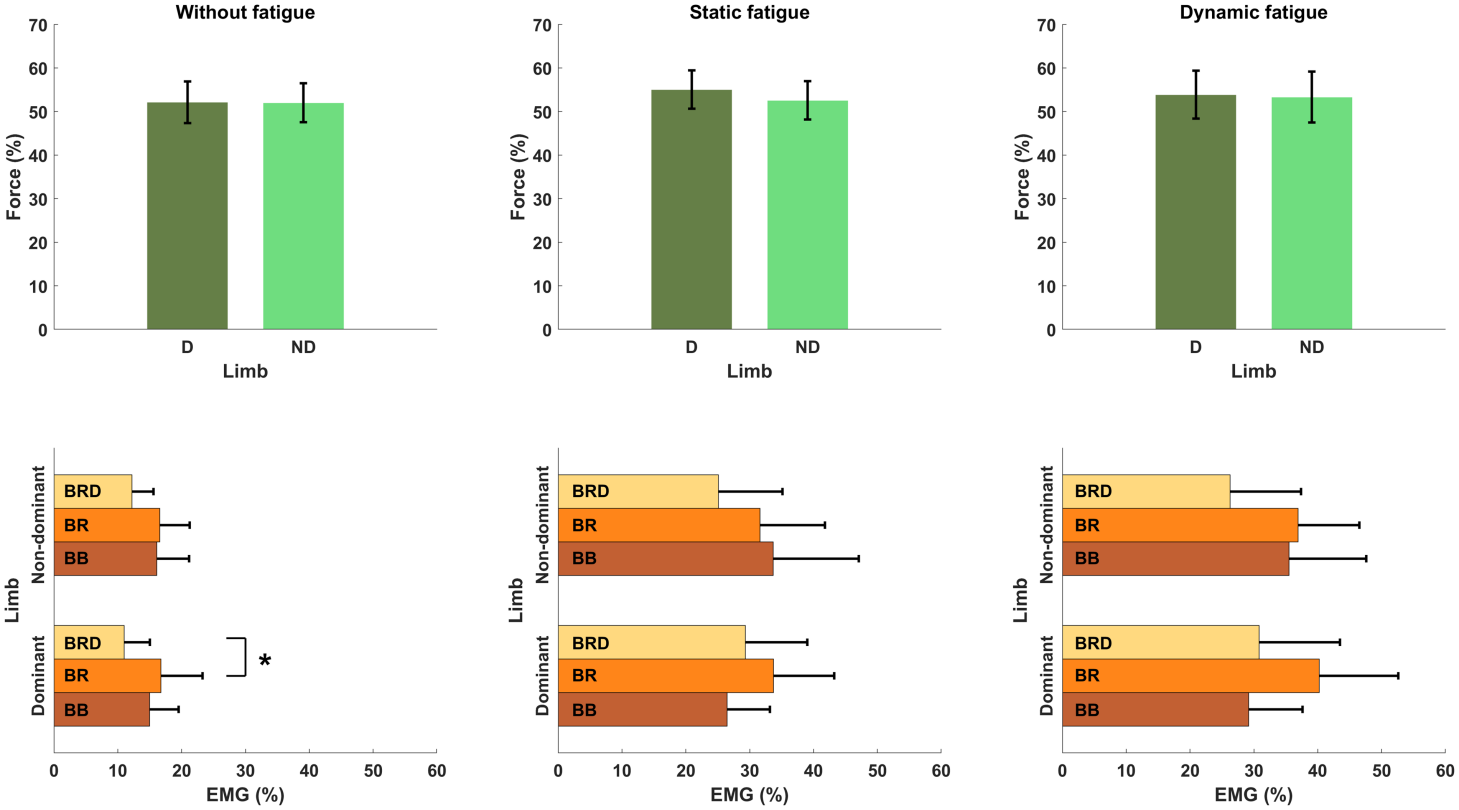
Structure of motor synergies in different types of fatigue protocol in elbow joints in force production task. D, dominant limb; ND, non-dominant limb; BB, biceps Brachii; BR, brachialis; BRD, brachioradialis.* - *p*<0.05.

To evaluate motor synergies on the upper level of applied hierarchy control, we analyzed forces produced by dominant and non-dominant limbs simultaneously. Higher magnitudes of force were generated by the dominant limb after fatigue. This trend was more observable after static type of fatigue protocol. However, the indicated difference between the limb forces and committed error by participants (despite fatigue) did not affect the force production task. On the lower level of the hierarchy in muscle activity we observed a distinguishable contribution of single muscle activity (Figure 6). Before applying the fatigue protocol, we found similar synergies in both upper limbs. The BR muscle was most involved in the execution of the task. The level of activity of all engaged muscles was similar, except BRD muscle in the dominant limb. BRD activity was significantly lower in relation to the strongest BR [*F*_(5,72)_ = 4.333, *p* = 0.02, *η*_p_^2^ = 0.194]. Under fatigue conditions, our analysis revealed both an increase in EMG signal and a completely new pattern of muscle involvement. After static fatigue, the weakest BRD in no fatigue condition surpasses the activity of BB in the dominant limb. In the non-dominant limb, the BB had greater magnitude compared to BR muscle. These changes in contribution are thought to cause changes in force, where after static fatigue non-dominant limb generated slightly lower magnitudes of force. Furthermore, after dynamic conditions, we observe a lower magnitude of BB activity in comparison to BRD in the dominant limb. These sophisticated changes in muscle activity are hard to observe in force data and do not appear to affect the force distribution in the upper level of the hierarchy. Hence, the one-way ANOVA analysis did not show significant differences between examined muscles after different types of fatigue. Different fatigue protocols caused changes in muscle activity contribution, especially in the non-dominant limb.

### 3.3 Effects of fatigue on wrist-elbow motor synergies

**Figure 7:**
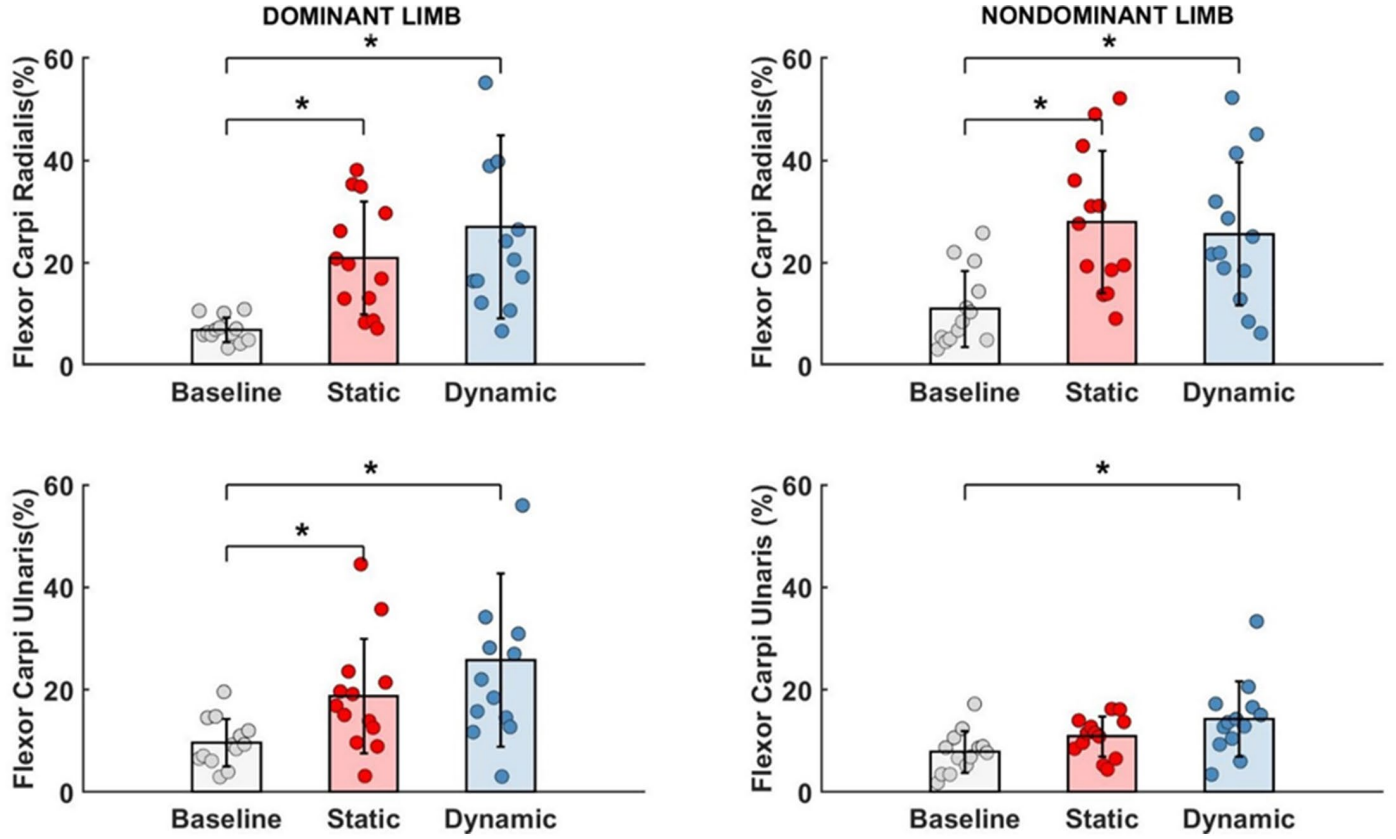
Significant impact of different types of fatigue on wrist flexors.* - *p*<0.05.

According to our Hypothesis #4 and using potential additional degrees of freedom by CNS, the EMG activity of wrist flexors was investigated (Figure 7).

**Figure 8:**
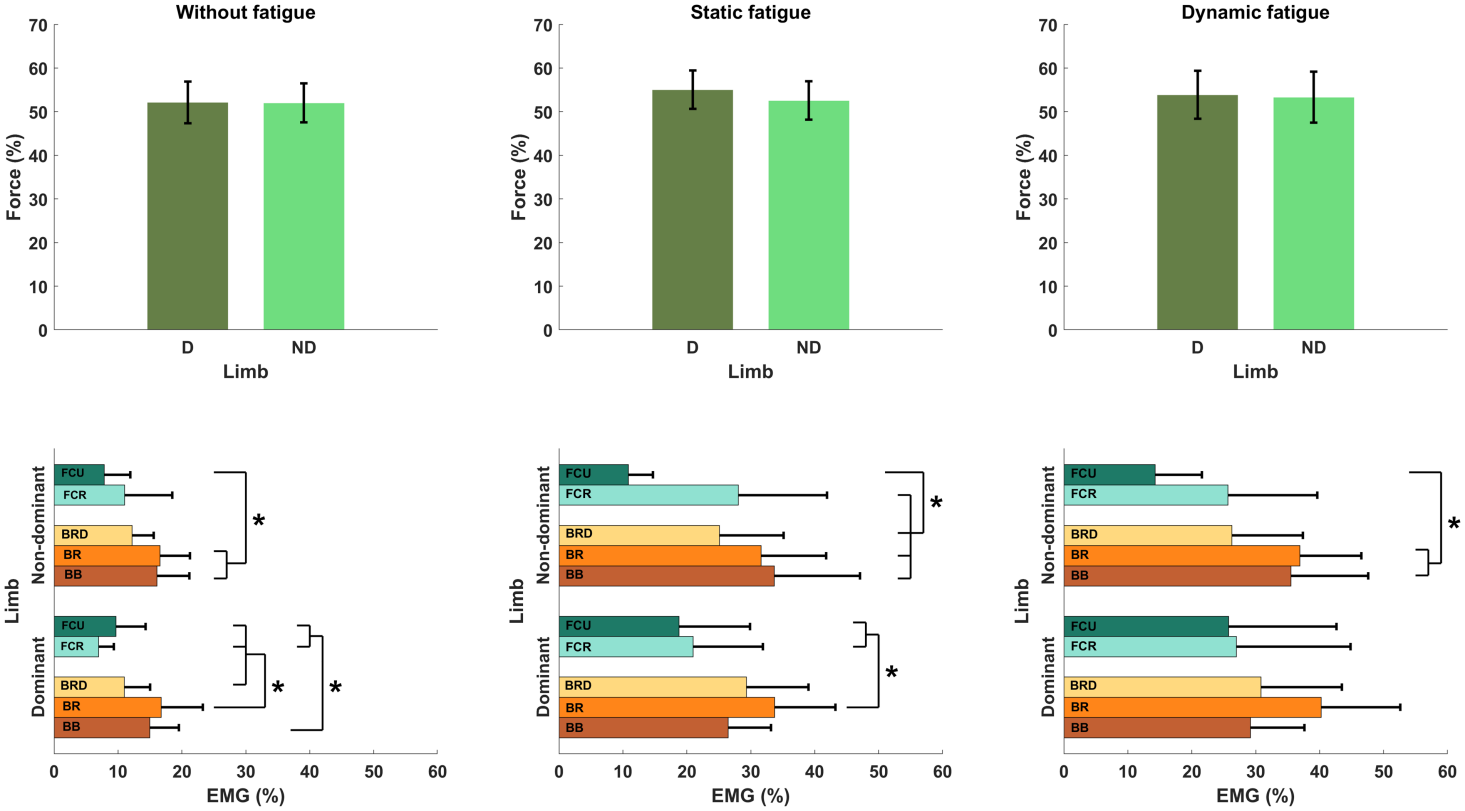
Structure of motor synergies in different types of fatigue include elbows and wrist flexors. D, dominant limb; ND, non-dominant limb; BB, biceps Brachii; BR, brachialis; BRD, brachioradialis; FCR, flexor carpi radialis; FCU, flexor carpi ulnaris.* - *p*<0.05.

For each measured wrist flexor, we identified a significant effect of fatigue. In dominant limb, EMG activity of FCR [*F*_(2,24)_ = 13.692, *p* < 0.001, *η*_p_^2^ = 0.509] and FCU [*F*_(2,24)_ = 12.447, *p* < 0.001, *η*_p_^2^ = 0.509] increased significantly by more than 15% after both measured fatigue types. In FCR, the dynamic condition of fatigue (*p* < 0.001) was more difficult for participants (required higher activation level) than a static condition (*p* = 0.005), where EMG activity was smaller, however there is no significant differences between the fatigue types (*p* > 0.05). This same pattern was observed in FCU of the dominant limb. Dynamic condition of fatigue (*p* < 0.001) resulted in higher EMG activity than in static condition (*p* = 0.029). In non-dominant limb and FCR EMG activity [*F*_(2,24)_ = 11.773, *p* < 0.001, *η*_p_^2^ = 0.495] we observed inverse significant trends. Static condition of fatigue showed a stronger effect with higher EMG activity (*p* < 0.001) than in dynamic condition (*p* = 0.002) in relation to the baseline level. In non-dominant limb FCU [*F*_(2,24)_ = 8.465, *p* = 0.002, *η*_p_^2^ = 0.414] the effect of fatigue was only observed after the dynamic fatigue condition (*p* = 0.001). The mentioned increase in wrist flexors EMG activity resulted in significant changes (Table 2) in the contribution of all muscles in the muscle synergies (Figure 8). In general, during the baseline condition without applying fatigue, we report a significant difference between elbow and wrist flexors in both limbs. In the dominant limb, both analyzed wrist flexors were characterized by relatively small EMG activity. In the non-dominant limb, we found the opposite FCR activity was higher than FCU. Then a static protocol of fatigue resulted in increased FCR muscle activity greater than 100% in both the dominant and non-dominant limb. Similar observation was made to FCU, but only in the dominant limb. In the non-dominant limb, we reported no significant increase. In relation to the baseline measurement, in the non-dominant limb, we reported higher activity of FCR compared to BRD. In the dominant limb, the activity of BR was significantly higher than all other analyzed muscles. In the dynamic type of fatigue, this difference disappeared. The wrist flexors achieved almost the same level of activity as BB and BRD in the dominant limb. In the non-dominant limb, similarly to the static condition, the activity of the FCU was significantly smaller than the BB and BR EMG signal. The activity of FCR was almost the same as BRD muscle.

**Table 2:** Main effects of one-factor ANOVA analysis of differences on expanded muscle activation space under different types of fatigue (elbow and wrist flexors).

Dominant limb			Non-dominant limb		
Main effect	Differences between muscles		Main effect	Differences between muscles	
No fatigue					
*F*_(4,60)_ = 9.654	BR vs. BRD	*p* = 0.022	*F*_(4,60)_ = 6.562	BB vs. FCU	*p* = 0.001
*p* < 0.001	BR vs. FCR	*p* < 0.001	*p* < 0.001	BR vs. FCU	*p* < 0.001
η_p_^2^ = 0.392	BR vs. FCU	*p* = 0.002	*η*_p_^2^ = 0.304		
	BB vs. FCR	*p* < 0.001			
	BB vs. FCU	*p* = 0.047			
Static fatigue					
*F*_(4,60)_ = 5.097	BR vs. FCR	*p* = 0.014	*F*_(4,60)_ = 8.926	BB vs. FCU	*p* < 0.001
*p* = 0.001	BR vs. FCU	*p* = 0.002	*p* < 0.001	BR vs. FCU	*p* < 0.001
*η*_p_^2^ = 0.254			*η*_p_^2^ = 0.373	BRD vs. FCU	*p* = 0.014
				FCR vs. FCU	*p* = 0.002
Dynamic fatigue					
			*F*_(4,60)_ = 8.865	BB vs. FCU	*p* < 0.001
			*p* < 0.001	BR vs. FCU	*p* < 0.001
			*η*_p_^2^ = 0.371		

*ηp2, effect size explained with partial eta-square; BB, biceps brachii; BR, brachialis; BRD, brachioradialis; FCR, flexor carpi radialis; FCU, flexor carpi ulnaris.*

## 4 Discussion

In this study, the participants were asked to perform the simultaneous force production task in isometric flexion of both elbow joints, before and after fatiguing protocol. We expected to see different effects of the two different fatigue protocols on the synergetic work of the upper limbs at two different levels of analysis: force level (forces produced by limbs) and muscle activity level. As predicted by Hypothesis #1, despite the fatigue factor, participants were able to perform force production task successfully. Our results demonstrate an increase in EMG data and produced forces, which has no effect on motor task performance. Fatigue had a different impact on various levels of hierarchical control of synergies. As mentioned above, the upper level of hierarchy control (level of forces) remains unchanged, whereas on the lower level (muscle activity) we observed different organization of motor synergies. Furthermore, we reported additional activity of wrist flexors and their contribution in forming synergies, as predicted by Hypothesis #3. We found no significant effects of different types of fatigue on the force production task, but observed their impact on examined muscle activity. Therefore, Hypothesis #2 was disproven.

One of the main effects of fatigue is a decrease in the ability of muscle fibers to generate force. In the literature, the most widely used index of fatigue is the level of decreased force with prolonged maximal voluntary contraction of involved muscles in the MVC task. Plenty of authors, based on above mentioned index of fatigue, observed a significant decrease in the MVC task during various activities performed by upper limbs: finger force production task (Danion et al., 2000, 2001; Danna-Dos Santos et al., 2010), sawing (Cowley et al., 2014), wrist extension (Huysmans et al., 2008), reaching (Fuller et al., 2009) or pointing movements (Cantú et al., 2014). In contrast, our study did not observe a significant drop in MVC force. Our study also reported a decrease in generated forces during fatigue tasks at high intensity (85% of MVC), but we used these results for the determination of the effectiveness of the applied fatigue protocols. In contrast to the studies listed above, as well as a proven decrease in the MVC value in the elbow muscles (Hunter and Enoka, 2001; Semmler et al., 2007; Missenard et al., 2008a), we did not analyze in our study the drop in MVC after fatigue protocol. Note, that the main task was a force production task at a relatively low intensity (30% of the MVC task). This methodology was adopted from Madeleine et al. (2002), where the continuous as well as intermittent movements were evaluated. Despite of the different design of our study, we also reported a lack of statistically significant outcomes in mean forces produced by upper limbs. In the main task, the aim was not to assess the drop in the MVC force magnitude but rather to evaluate the execution of simultaneous force production of given intensity with both upper limbs. For this reason, the committed errors of the participants were analyzed (in the overestimation or underestimation aspect). In the presented results, performing a fatigue protocol caused increasing force errors, however these results were not statistically significant. These result contrast with previous reports in the literature, where fatigue leads to a significant reduction in the accuracy of performed tasks (Singh et al., 2010, 2012, 2013; Park et al., 2012). However, in the aforementioned studies, authors analyzed only control in smaller elements of the movement system (finger movements) and set the task intensity at a different range than in the our work. Increased magnitudes of force errors after fatigue were also reported by other studies (Missenard et al., 2008b; Chen et al., 2012), where the movement kinematics of upper limbs were assessed. Nevertheless, at the level of muscle activation, we reported a significant impact of fatigue, independently of type, for each analyzed elbow flexor muscle. The EMG activity was significantly higher after applying the fatigue protocol. The obtained results are consistent with our earlier predictions. In the literature, regardless of the type of fatigue, other authors have obtained similar results for static (Hunter and Enoka, 2001; Kattla and Lowery, 2010; Yoon et al., 2013) and dynamic conditions (Post et al., 2008; Fuller et al., 2009; Cantú et al., 2014).

The lack of differences between the type of applied fatigue and no differences in force errors turned out to be unexpected and inconsistent with our hypotheses. Despite meaningful differences in the nature of applied type of fatigue (Genaidy et al., 1990), other authors reported significant differences between static and dynamic conditions (Clarke, 1962; Lanza et al., 2004; Klass et al., 2005). Furthermore, studies have shown increased muscle activity following dynamic fatigue conditions such as holding an object compared to static conditions. The increase in the amplitude of the muscle activity after fatigue could be the result of the process of increasing the recruitment of individual motor units and changes in their frequencies, which is intended to compensate for the decreased ability of muscle fiber to generate the force (Bigland-Ritchie et al., 1986; Garland et al., 1994; Riley et al., 2008). On the opposite side, these results are in oppositions of other studies, where significant differences between fatigue conditions were not reported (Christensen et al., 1995; Sogaard, 1995). However, despite the lack of differences in EMG outcomes, it was suggested that measured aspects of fatigue are very sensitive to the used method of analysis (Arias et al., 2015; Pethick et al., 2019), where using a more sophisticated method of analysis revealed a significant difference between static and dynamic condition of fatigue.

From the other side, we reported the behavior of CNS to ensures stability of executing movement. In this study, participants successfully performed a force production task after fatigue of different types. Despite of similar tendencies of changes at two different levels of hierarchical movement control, we did not observe a significant difference between fatigue caused by different type of muscle contraction. Perhaps, at the higher level of hierarchy control, the level of intensity of the force production task was too small and relatively easy for participants to show significant effect of fatigue of fatigue in the bimanual movement. Nevertheless, at the lower level of hierarchy control, we observed a strong significant difference in the EMG activity of each of the measured muscles. These results support other author’s conclusions about problems with performance stabilization on levels of the analyzed neural hierarchy of movement (Gorniak et al., 2007a, 2007b). It seems that for CNS, stabilization of movement performance on the upper level (successful execution of the specific movement) is a priority and the lower level of the hierarchy is characterized by a greater magnitude of variability (Gorniak et al., 2009; Reschechtko and Latash, 2017). Furthermore, we observed significant differences in the activity of the wrist flexor muscles for an isometric flexion force production task after fatigue in the elbow joints (both dominant and non-dominant limb). Similarly, for elbow flexor muscles, there was a significant increase in muscle activity. The reason behind the significant increase in wrist flexor EMG activity during an isometric force production tasks in the elbow joint is still not fully understood. There are few theoretical concepts in motor control that may explain the above phenomena: First, it could be the rule of motor abundance, which speaks not about a characteristic, single way of performing movement, but of a group (family) of solutions of performing it effectively (Gelfand and Latash, 1998; Latash, 2012). According to this rule, CNS fatigue results in the use of additional degrees of freedom of movement (other structures of the musculoskeletal system allow to perform the examined motor task effectively). If the elbow flexors were not able to perform the main task, wrist flexors were activated to ensure task completion. On the other hand, the synergistic action of wrist muscles could be responsible for a mechanical connection of the elbow and wrist joint and their control by the CNS. Dounskaia et al. (1998) refer to numerous studies which hypothesize that biomechanical aspects cause different movement control for the aforementioned joints. The execution of movement in the elbow joint is controlled similarly in each of its phases, which confirms the well-known theory of motion in a single joint (the activity of agonists responsible for starting the movement and antagonists for its inhibition). However, the muscle activity generated by the muscles surrounding the elbow joint also affects the generation of movements (moment of force) for the wrist joints (Putnam, 1993). The results of Dounskaia et al. (1998) corroborate the above hypothesis and draw attention to the function of passive elements of the musculoskeletal system and the viscoelastic properties of muscle tissue. For this reason, movement control in the wrist joints tends to be more complicated for the CNS than in the elbow joints. From a neurophysiological perspective, the control of flexion movements in the upper limb could be related to the spinal flexor reflex afferents, which are based on the action of secondary fibers in the muscle spindles causing the flexion reflex at the same limb. Due to the transmission of signals at many levels of the spinal cord, this reflex usually exists in the entire limb. The effects of the functioning of long descending paths of the spinal cord allow for its use during voluntary movements. A final concept capable of explaining the synergistic action of the aforementioned joints is the concept of the superiority of one joint over another in the process of movement control. The muscle activity of wrist flexors is preceded by the activity of elbow flexors, which significantly affects the movement execution in the wrists (Cooke and Virji-Babul, 1995). Similar conclusions were made in the work of Latash et al. (1995), where the elbow joint performs a leading role in the main task while the wrist joints counteract the negative effects of arm movement and allow for correct task performance. Despite many concepts trying to explain the movement control in the elbow and wrist joint, it is still not fully understood, how the coordination (synergy) is functioning.

In conclusion, our study demonstrated the effects of different types of fatigue on force production tasks in the elbow joints, with significant changes observed only at the lower levels of the neural hierarchy controlling motor synergies. This suggests that the CNS prioritizes adjustments at lower levels of neural control rather than altering overall movement performance. Despite using different fatigue protocols, we found no significant differences between them, likely due to the absence of EMG data from antagonist muscles, which may have limited the interpretation of key findings such as force errors and muscle synergy changes. Furthermore, relatively small number of participants may be considered as another limitation of our study. Due to the complexity of the research protocol and its nature, where fatigue was dedicated only to men (efforts of high intensity and relatively short duration) (Hunter and Enoka, 2001; Hunter, 2009; Hunter, 2018), the quantity of participants was chosen based on the literature review in this aspect. From our perspective, the results of this study may have potential in other fields than motor control such as sport sciences or clinical implications. In different sports disciplines, the motor synergies after fatigue could help with introducing new training protocols which could allow for better preparation of athletes to the high level sport performance. From the other hand, the clinicians can modify and adjust specific rehabilitation programs and therapies for different groups of patients with upper limb dysfunctions. For future studies, we aim to use more advanced fatigue assessment methods, such as Fourier and Wavelet transformations, to increase sensitivity to fatigue effects. Additionally, we plan to explore alternative synergy definitions (D’Avella et al., 2003; Latash et al., 2007; Latash, 2021) and analyze fatigue effects within the M-mode space using dedicated synergy indices and the uncontrolled manifold hypothesis (Scholz and Schöner, 1999; Latash, 2021).

## Data Availability

The raw data supporting the conclusions of this article will be made available by the authors, without undue reservation.

## References

[ref1] Alix-Fages C. Grgic J. Jiménez-Martínez P. Baz-Valle E. Balsalobre-Fernández C. (2023). Effects of mental fatigue on strength endurance: a systematic review and Meta-analysis. Mot. Control. 27, 442–461. doi: 10.1123/mc.2022-0051, PMID: 36509089

[ref2] Al-Mulla M. R. Sepulveda F. Colley M. (2011). A review of non-invasive techniques to detect and predict localized muscle fatigue. Sensors 11, 3545–3594. doi: 10.3390/s110403545, PMID: 22163810 PMC3231314

[ref3] Arias P. Robles-García V. Corral-Bergantiños Y. Madrid A. Espinosa N. Valls-Solé J.. (2015). Central fatigue induced by short-lasting finger tapping and isometric tasks: a study of silent periods evoked at spinal and supraspinal levels. Neuroscience 305, 316–327. doi: 10.1016/j.neuroscience.2015.07.081, PMID: 26241342

[ref4] Behm D. G. Alizadeh S. Hadjizedah Anvar S. Hanlon C. Ramsay E. Mahmoud M. M. I.. (2021). Non-local muscle fatigue effects on muscle strength, Power, and endurance in healthy individuals: a systematic review with Meta-analysis. Sport Med 51, 1893–1907. doi: 10.1007/s40279-021-01456-3, PMID: 33818751

[ref5] Behrens M. Gube M. Chaabene H. Prieske O. Zenon A. Broscheid K. C.. (2023). Fatigue and human performance: an updated framework. Sport Med 53, 7–31. doi: 10.1007/s40279-022-01748-2, PMID: 36258141 PMC9807493

[ref6] Bernstein N. A. (1967) The co-ordination and regulation of movements. Oxford Pergamon Press.

[ref7] Bigland-Ritchie B. Furbush F. Woods J. J. (1986). Fatigue of intermittent submaximal voluntary contractions: central and peripheral factors. J. Appl. Physiol. 61, 421–429. doi: 10.1152/jappl.1986.61.2.421, PMID: 3745035

[ref8] Bonnard M. Oddsson L. Sirin A. V. Thorstennson A. (1994). Different strategies to compensate for the effect of fatigue revealed by neuromuscular adaptation processes in humans. Neurosci. Lett. 166, 101–105. doi: 10.1016/0304-3940(94)90850-8, PMID: 8190349

[ref9] Cantú H. Emery K. Côté J. N. (2014). Effects of additional external weight on posture and movement adaptations to fatigue induced by a repetitive pointing task. Hum. Mov. Sci. 35, 1–16. doi: 10.1016/j.humov.2014.02.003, PMID: 24786737

[ref10] Chen Y. C. Yang J. F. Hwang I. S. Baldissera F. (2012). Global effect on multi-segment physiological tremors due to localized fatiguing contraction. Eur. J. Appl. Physiol. 112, 899–910. doi: 10.1007/s00421-011-2044-7, PMID: 21688156

[ref11] Christensen H. Søgaard K. Jensen B. R. Finsen L. Sjøgaard G. (1995). Intramuscular and surface EMG power spectrum from dynamic and static contractions. J. Electromyogr. Kinesiol. 5, 27–36. doi: 10.1016/S1050-6411(99)80003-0, PMID: 20719634

[ref12] Clarke D. H. (1962). Strength recovery from static and dynamic muscular fatigue. Res Q Am Assoc Heal Phys Educ Recreat 33, 349–355. doi: 10.1080/10671188.1962.10616463, PMID: 39659294

[ref13] Cooke J. D. Virji-Babul N. (1995). Reprogramming of muscle activation patterns at the wrist in compensation for elbow reaction torques during planar two-joint arm movements. Exp. Brain Res. 106, 169–176. doi: 10.1007/BF00241366, PMID: 8542973

[ref14] Côté J. N. Mathieu P. A. Levin M. F. Feldman A. G. (2002). Movement reorganization to compensate for fatigue during sawing. Exp. Brain Res. 146, 394–398. doi: 10.1007/s00221-002-1186-6, PMID: 12232697

[ref15] Cowley J. C. Dingwell J. B. Gates D. H. (2014). Effects of local and widespread muscle fatigue on movement timing. Exp. Brain Res. 232, 3939–3948. doi: 10.1007/s00221-014-4020-z, PMID: 25183157 PMC4241184

[ref16] Cowley J. C. Gates D. H. (2017). Inter-joint coordination changes during and after muscle fatigue. Hum. Mov. Sci. 56, 109–118. doi: 10.1016/j.humov.2017.10.015, PMID: 29121490

[ref17] D’Avella A. Saltiel P. Bizzi E. (2003). Combinations of muscle synergies in the construction of a natural motor behavior. Nat. Neurosci. 6, 300–308. doi: 10.1038/nn1010, PMID: 12563264

[ref18] Danion F. Latash M. L. Li Z. M. Zatsiorsky V. M. (2000). The effect of fatigue on multifinger coordination in force production tasks in humans. J. Physiol. 523, 523–532. doi: 10.1111/j.1469-7793.2000.00523.x, PMID: 10699094 PMC2269799

[ref19] Danion F. Latash M. L. Li Z. M. Zatsiorsky V. M. (2001). The effect of a fatiguing exercise by the index finger on single-and multi-finger force production tasks. Exp. Brain Res. 138, 322–329. doi: 10.1007/s002210100698, PMID: 11460770 PMC2830622

[ref20] Danna-Dos Santos A. Poston B. Jesunathadas M. Bobich L. R. Hamm T. M. Santello M. (2010). Influence of fatigue on hand muscle coordination and EMG-EMG coherence during three-digit grasping. J. Neurophysiol. 104, 3576–3587. doi: 10.1152/jn.00583.2010, PMID: 20926609 PMC3007653

[ref21] Dounskaia N. V. Swinnen S. P. Walter C. B. Spaepen A. J. Verschueren S. M. P. (1998). Hierarchical control of different elbow-wrist coordination patterns. Exp. Brain Res. 121, 239–254. doi: 10.1007/s002210050457, PMID: 9746130

[ref22] Enoka R. M. Baudry S. Rudroff T. Farina D. Klass M. Duchateau J. (2011). Unraveling the neurophysiology of muscle fatigue. J. Electromyogr. Kinesiol. 21, 208–219. doi: 10.1016/j.jelekin.2010.10.006, PMID: 21071242

[ref23] Enoka R. M. Duchateau J. (2008). Muscle fatigue: what, why and how it influences muscle function. J. Physiol. 586, 11–23. doi: 10.1113/jphysiol.2007.139477, PMID: 17702815 PMC2375565

[ref24] Enoka R. Duchateau J. (2016). Translating fatigue to human performance. Med. Sci. Sports Exerc. 48, 2228–2238. doi: 10.1249/MSS.0000000000000929, PMID: 27015386 PMC5035715

[ref25] Enoka R. M. Stuart D. G. (1992). Neurobiology of muscle fatigue. J. Appl. Physiol. 72, 1631–1648. doi: 10.1152/jappl.1992.72.5.1631, PMID: 1601767

[ref26] Evans N. (2007). Bodybuilding anatomy. Human Kinetics.

[ref27] Forestier N. Nougier V. (1998). The effects of muscular fatigue on the coordination of a multijoint movement in human. Neurosci. Lett. 252, 187–190. doi: 10.1016/S0304-3940(98)00584-9, PMID: 9739992

[ref28] Fuller J. R. Lomond K. V. Fung J. Côté J. N. (2009). Posture-movement changes following repetitive motion-induced shoulder muscle fatigue. J. Electromyogr. Kinesiol. 19, 1043–1052. doi: 10.1016/j.jelekin.2008.10.009, PMID: 19091598

[ref29] Gandevia S. C. (2001). Spinal and supraspinal factors in human muscle fatigue. Physiol. Rev. 81, 1725–1789. doi: 10.1152/physrev.2001.81.4.1725, PMID: 11581501

[ref30] Garland S. J. Enoka R. M. Serrano L. P. Robinson G. A. (1994). Behavior of motor units in human biceps brachii during a submaximal fatiguing contraction. J. Appl. Physiol. 76, 2411–2419. doi: 10.1152/jappl.1994.76.6.2411, PMID: 7928865

[ref31] Gates D. H. Dingwell J. B. (2008). The effects of neuromuscular fatigue on task performance during repetitive goal-directed movements. Exp. Brain Res. 187, 573–585. doi: 10.1007/s00221-008-1326-8, PMID: 18327575 PMC2825378

[ref32] Gelfand I. M. Latash M. L. (1998). On the problem of adequate language in motor control. Mot. Control. 2, 306–313. 10.1123/mcj.2.4.306 9758883

[ref33] Genaidy A. M. Houshyar A. Asfour S. S. (1990). Physiological and psychophysical responses to static, dynamic and combined arm tasks. Appl. Ergon. 21, 63–67. doi: 10.1016/0003-6870(90)90075-9, PMID: 15676761

[ref34] Gorniak S. L. Zatsiorsky V. M. Latash M. L. (2007a). Emerging and disappearing synergies in a hierarchically controlled system. Exp. Brain Res. 183, 259–270. doi: 10.1007/s00221-007-1042-9, PMID: 17703288 PMC2827035

[ref35] Gorniak S. L. Zatsiorsky V. M. Latash M. L. (2007b). Hierarchies of synergies: an example of two-hand, multi-finger tasks. Exp. Brain Res. 179, 167–180. doi: 10.1007/s00221-006-0777-z, PMID: 17103206 PMC1859846

[ref36] Gorniak S. L. Zatsiorsky V. M. Latash M. L. (2009). Hierarchical control of static prehension: II. Multi-digit synergies. Exp Brain Res 194, 1–15. doi: 10.1007/s00221-008-1663-7, PMID: 19048236 PMC2656415

[ref37] Hajiloo B. Anbarian M. Esmaeili H. Mirzapour M. (2020). The effects of fatigue on synergy of selected lower limb muscles during running. J. Biomech. 103:109692. doi: 10.1016/j.jbiomech.2020.109692, PMID: 32151383

[ref38] Hasanbarani F. Latash M. L. (2020). Performance-stabilizing synergies in a complex motor skill: analysis based on the uncontrolled manifold hypothesis. Mot. Control. 24, 238–252. doi: 10.1123/mc.2019-0049, PMID: 31914422

[ref39] Hermens H. J. Freriks B. Disselhorst-Klug C. Rau G. (2000). Development of recommendations for SEMG sensors and sensor placement procedures. J. Electromyogr. Kinesiol. 10, 361–374. doi: 10.1016/S1050-6411(00)00027-4, PMID: 11018445

[ref40] Huffenus A.-F. Amarantini D. Forestier N. (2006). Effects of distal and proximal arm muscles fatigue on multi-joint movement organization. Exp. Brain Res. 170, 438–447. doi: 10.1007/s00221-005-0227-3, PMID: 16369793

[ref41] Hunter S. K. (2009). Sex differences and mechanisms of task-specific muscle fatigue. Exerc. Sport Sci. Rev. 37, 113–122. doi: 10.1097/JES.0b013e3181aa63e2, PMID: 19550202 PMC2909485

[ref42] Hunter S. K. (2018). Performance fatigability: mechanisms and task specificity. Cold Spring Harb. Perspect. Med. 8, 1–21. doi: 10.1101/cshperspect.a029728, PMID: 28507192 PMC6027928

[ref43] Hunter S. K. Duchateau J. Enoka R. M. (2004). Muscle fatigue and the mechanisms of task failure. Exerc. Sport Sci. Rev. 32, 44–49. doi: 10.1097/00003677-200404000-00002, PMID: 15064647

[ref44] Hunter S. K. Enoka R. M. (2001). Sex differences in the fatigability of arm muscles depends on absolute force during isometric contractions. J. Appl. Physiol. 91, 2686–2694. doi: 10.1152/jappl.2001.91.6.2686, PMID: 11717235

[ref45] Huysmans M. A. Hoozemans M. J. M. van der Beek A. J. de Looze M. P. van Dieën J. H. (2008). Fatigue effects on tracking performance and muscle activity. J. Electromyogr. Kinesiol. 18, 410–419. doi: 10.1016/j.jelekin.2006.11.003, PMID: 17208457

[ref46] Jaric S. Blesic S. Milanovic S. Radovanovic S. Ljubisavljevic M. Anastasijevic R. (1999). Changes in movement final position associated with agonist and antagonist muscle fatigue. Eur. J. Appl. Physiol. Occup. Physiol. 80, 467–471. doi: 10.1007/s004210050619, PMID: 10502081

[ref47] Jones F. P. Hanson J. A. (1971). Fatigue effects on patterns of movement. Ergonomics 14, 391–410. doi: 10.1080/00140137108931259, PMID: 5096454

[ref48] Kattla S. Lowery M. M. (2010). Fatigue related changes in electromyographic coherence between synergistic hand muscles. Exp. Brain Res. 202, 89–99. doi: 10.1007/s00221-009-2110-0, PMID: 20012600

[ref49] Klass M. Baudry S. Duchateau J. (2005). Aging does not affect voluntary activation of the ankle dorsiflexors during isometric, concentric, and eccentric contractions. J. Appl. Physiol. 99, 31–38. doi: 10.1152/japplphysiol.01426.2004, PMID: 15705734

[ref50] Kluger B. M. Krupp L. B. Enoka R. M. (2013). Fatigue and fatigability in neurologic illnesses: proposal for a unified taxonomy. Neurology 80, 409–416. doi: 10.1212/WNL.0b013e31827f07be, PMID: 23339207 PMC3589241

[ref51] Krüger R. L. Aboodarda S. J. Samozino P. Rice C. L. Millet G. Y. (2018). Isometric versus dynamic measurements of fatigue: does age matter? A meta-analysis. Med. Sci. Sports Exerc. 50, 2132–2144. doi: 10.1249/MSS.0000000000001666, PMID: 29787475

[ref52] Lanza I. R. Russ D. W. Kent-Braun J. A. (2004). Age-related enhancement of fatigue resistance is evident in men during both isometric and dynamic tasks. J. Appl. Physiol. 97, 967–975. doi: 10.1152/japplphysiol.01351.2003, PMID: 15145914

[ref53] Latash M. (2012). Fundamentals of motor control. Elsevier: Academic Press.

[ref54] Latash M. L. (2021). Understanding and synergy: a single concept at different levels of analysis? Front. Syst. Neurosci. 15, 1–10. doi: 10.3389/fnsys.2021.735406, PMID: 34867220 PMC8636674

[ref55] Latash M. L. Aruin A. S. Shapiro M. B. (1995). The relation between posture and movement: a study of a simple synergy in a two-joint task. Hum. Mov. Sci. 14, 79–107. doi: 10.1016/0167-9457(94)00046-H

[ref56] Latash M. L. Gorniak S. Zatsiorsky V. M. (2008). Hierarchies of synergies in human movements. Kinesiology 40, 29–38. 20354578 PMC2846665

[ref57] Latash M. L. Madarshahian S. Ricotta J. M. (2023). Intramuscle synergies: their place in the neural control hierarchy. Mot. Control. 27, 402–441. doi: 10.1123/mc.2022-0094, PMID: 36543175

[ref58] Latash M. L. Scholz J. P. Schöner G. (2007). Toward a new theory of motor synergies. Mot. Control. 11, 276–308. doi: 10.1123/mcj.11.3.276 17715460

[ref59] Latash M. Zatsiorsky V. M. (2016). Biomechanics and motor control: Defining central concepts. Elsevier: Academic Press.

[ref60] Lucidi C. A. Lehman S. L. (1992). Adaptation to fatigue of long duration in human wrist movements. J. Appl. Physiol. 73, 2596–2603. doi: 10.1152/jappl.1992.73.6.2596, PMID: 1490975

[ref61] Madeleine P. Jørgensen L. V. Søgaard K. Arendt-Nielsen L. Sjogaard G. (2002). Development of muscle fatigue as assessed by electromyography and mechanomyography during continuous and intermittent low-force contractions: effects of the feedback mode. Eur. J. Appl. Physiol. 87, 28–37. doi: 10.1007/s00421-002-0578-4, PMID: 12012073

[ref62] Maluf K. S. Enoka R. M. (2005). Task failure during fatiguing contractions performed by humans. J. Appl. Physiol. 99, 389–396. doi: 10.1152/japplphysiol.00207.2005, PMID: 16020434

[ref63] Missenard O. Mottet D. Perrey S. (2008a). Muscular fatigue increases signal-dependent noise during isometric force production. Neurosci. Lett. 437, 154–157. doi: 10.1016/j.neulet.2008.03.090, PMID: 18440146

[ref64] Missenard O. Mottet D. Perrey S. (2008b). The role of cocontraction in the impairment of movement accuracy with fatigue. Exp. Brain Res. 185, 151–156. doi: 10.1007/s00221-007-1264-x, PMID: 18205000

[ref65] Nussbaum M. A. (2001). Static and dynamic myoelectric measures of shoulder muscle fatigue during intermittent dynamic exertions of low to moderate intensity. Eur. J. Appl. Physiol. 85, 299–309. doi: 10.1007/s004210100454, PMID: 11560084

[ref66] Oldfield R. C. (1971). The assessment and analysis of handedness: the Edinburgh inventory. Neuropsychologia 9, 97–113. doi: 10.1016/0028-3932(71)90067-4 5146491

[ref67] Ortega-Auriol P. A. Besier T. F. Byblow W. D. McMorland A. J. C. (2018). Fatigue influences the recruitment, but not structure, of muscle synergies. Front. Hum. Neurosci. 12, 1–12. doi: 10.3389/fnhum.2018.00217 29977197 PMC6021531

[ref68] Paris M. T. McNeil C. J. Power G. A. Rice C. L. Dalton B. H. (2022). Age-related performance fatigability: a comprehensive review of dynamic tasks. J. Appl. Physiol. 133, 850–866. doi: 10.1152/japplphysiol.00319.2022, PMID: 35952347

[ref69] Park J. Singh T. Zatsiorsky V. M. Latash M. L. (2012). Optimality versus variability: effect of fatigue in multi-finger redundant tasks. Exp. Brain Res. 216, 591–607. doi: 10.1007/s00221-011-2963-x, PMID: 22130781 PMC3623544

[ref70] Pawłowski M. Furmanek M. P. Sobota G. Marszałek W. Słomka K. Bacik B.. (2021). Number of trials necessary to apply analysis within the framework of the uncontrolled manifold hypothesis at different levels of hierarchical synergy control. J. Hum. Kinet. 76, 131–143. doi: 10.2478/hukin-2021-0005, PMID: 33603930 PMC7877275

[ref71] Pereira R. Mendel M. M. A. Schettino L. Machado M. Augusto-Silva P. (2013). Acute neuromuscular responses to a resistance exercise session performed using the Delorme and Oxford techniques. Hum Mov 14, 347–352. doi: 10.2478/humo-2013-0042, PMID: 39513035

[ref72] Perotto A. O. (2005). Anatomical guide for the electromyographer. Springfield, Illinois, USA: Charles C Thomas.

[ref73] Pethick J. Whiteaway K. Winter S. L. Burnley M. (2019). Prolonged depression of knee-extensor torque complexity following eccentric exercise. Exp. Physiol. 104, 100–111. doi: 10.1113/EP087295, PMID: 30485571

[ref74] Post M. Bayrak S. Kernell D. Zijdewind I. (2008). Contralateral muscle activity and fatigue in the human first dorsal interosseous muscle. J. Appl. Physiol. 105, 70–82. doi: 10.1152/japplphysiol.01298.2007, PMID: 18450978

[ref75] Putnam C. A. (1993). Sequential motions of body segments in striking and throwing skills: descriptions and explanations. J. Biomech. 26, 125–135. doi: 10.1016/0021-9290(93)90084-R 8505347

[ref76] Reschechtko S. Latash M. L. (2017). Stability of hand force production: I. Hand level control variables and multi-finger synergies. J. Neurophysiol. 118, 3152–3164. doi: 10.1152/jn.00485.2017 28904102 PMC5814709

[ref77] Riley Z. A. Maerz A. H. Litsey J. C. Enoka R. M. (2008). Motor unit recruitment in human biceps brachii during sustained voluntary contractions. J. Physiol. 586, 2183–2193. doi: 10.1113/jphysiol.2008.150698, PMID: 18292128 PMC2465192

[ref78] Scholz J. P. Schöner G. (1999). The uncontrolled manifold concept: identifying control variables for a functional task. Exp. Brain Res. 126, 289–306. doi: 10.1007/s002210050738, PMID: 10382616

[ref79] Semmler J. G. Tucker K. J. Allen T. J. Proske U. (2007). Eccentric exercise increases EMG amplitude and force fluctuations during submaximal contractions of elbow flexor muscles. J. Appl. Physiol. 103, 979–989. doi: 10.1152/japplphysiol.01310.2006, PMID: 17600154

[ref80] Singh T. SKM V. Zatsiorsky V. M. Latash M. L. (2010). Adaptive increase in force variance during fatigue in tasks with low redundancy. Neurosci. Lett. 485, 204–207. doi: 10.1016/j.neulet.2010.09.012, PMID: 20849913 PMC2956869

[ref81] Singh T. Zatsiorsky V. M. Latash M. L. (2012). Effects of fatigue on synergies in a hierarchical system. Hum. Mov. Sci. 31, 1379–1398. doi: 10.1016/j.humov.2012.06.008, PMID: 23182434 PMC3759811

[ref82] Singh T. Zatsiorsky V. M. Latash M. L. (2013). Contrasting effects of fatigue on multifinger coordination in young and older adults. J. Appl. Physiol. 115, 456–467. doi: 10.1152/japplphysiol.00375.2013, PMID: 23743395 PMC3742945

[ref83] Sogaard K. (1995). Motor unit recruitment pattern during low-level static and dynamic contractions. Muscle Nerve 18, 292–300. doi: 10.1002/mus.880180305, PMID: 7870106

[ref84] Turpin N. A. Guével A. Durand S. Hug F. (2011). Fatigue-related adaptations in muscle coordination during a cyclic exercise in humans. J. Exp. Biol. 214, 3305–3314. doi: 10.1242/jeb.057133, PMID: 21900479

[ref85] Vieira J. G. Sardeli A. V. Dias M. R. Filho J. E. Campos Y. Sant’Ana L.. (2022). Effects of resistance training to muscle failure on acute fatigue: a systematic review and meta-analysis. Sport Med 52, 1103–1125. doi: 10.1007/s40279-021-01602-x, PMID: 34881412

[ref86] Willardson J. M. Burkett L. N. (2005). A comparison of 3 different rest intervals on the exercise volume completed during a workout. J. Strength Cond. Res. 19, 23–26. doi: 10.1519/R-13853.1 15705039

[ref87] World Medical Association (2013). World medical association declaration of Helsinki: ethical principles for medical research involving human subjects. JAMA 310, 2191–2194. doi: 10.1001/jama.2013.281053, PMID: 24141714

[ref88] Yoon T. Schlinder-delap B. Hunter S. K. (2013). Fatigability and recovery of arm muscles with advanced age for dynamic and isometric contractions. Exp. Gerontol. 48, 259–268. doi: 10.1016/j.exger.2012.10.006, PMID: 23103238 PMC3557758

